# Detection of ubiquitinated huntingtin species in intracellular aggregates

**DOI:** 10.3389/fnmol.2015.00001

**Published:** 2015-01-28

**Authors:** Katrin Juenemann, Anne Wiemhoefer, Eric A. Reits

**Affiliations:** Department of Cell Biology and Histology, Academic Medical Center, University of AmsterdamAmsterdam, Netherlands

**Keywords:** Huntington’s disease, huntingtin, aggregation, formic acid, ubiquitination

## Abstract

Protein conformation diseases, including polyglutamine (polyQ) diseases, result from the accumulation and aggregation of misfolded proteins. Huntington’s disease (HD) is one of nine diseases caused by an expanded polyQ repeat within the affected protein and is hallmarked by intracellular inclusion bodies composed of aggregated N-terminal huntingtin (Htt) fragments and other sequestered proteins. Fluorescence microscopy and filter trap assay are conventional methods to study protein aggregates, but cannot be used to analyze the presence and levels of post-translational modifications of aggregated Htt such as ubiquitination. Ubiquitination of proteins can be a signal for degradation and intracellular localization, but also affects protein activity and protein-protein interactions. The function of ubiquitination relies on its mono- and polymeric isoforms attached to protein substrates. Studying the ubiquitination pattern of aggregated Htt fragments offers an important possibility to understand Htt degradation and aggregation processes within the cell. For the identification of aggregated Htt and its ubiquitinated species, solubilization of the cellular aggregates is mandatory. Here we describe methods to identify post-translational modifications such as ubiquitination of aggregated mutant Htt. This approach is specifically described for use with mammalian cell culture and is suitable to study other disease-related proteins prone to aggregate.

## Introduction

Various age-dependent neurodegenerative disorders, such as Huntington’s disease (HD), Parkinson’s disease (PD) or Alzheimer’s disease (AD), are hallmarked by aggregation of misfolded proteins in neurons with age (Ross and Tabrizi, 2011; Benilova et al., 2012; Irwin et al., 2013). HD is one of nine polyglutamine (polyQ) diseases caused by an expansion of a CAG trinucleotide repeat that encodes a polyQ tract in the huntingtin (Htt) protein that triggers its aggregation (Yamada et al., 2008). A neuropathological hallmark of HD is the presence of intracellular inclusion bodies composed of mutant Htt N-terminal fragments found in human postmortem brain, animal models, and cell culture models (DiFiglia et al., 1997; Gutekunst et al., 1999; Lunkes et al., 2002; Schilling et al., 2007; Juenemann et al., 2011). The idea that intracellular inclusions contain N-terminal fragments of Htt has been supported by immunological studies, where inclusions of HD patients could be stained by Htt antibodies against the N-terminal part of Htt but not antibodies against the C-terminal part (Lunkes et al., 2002). Moreover, pathological changes are accelerated in HD mouse models overexpressing N-terminal fragments of mutant Htt compared to those with full-length mutant Htt (Mangiarini et al., 1996; Hodgson et al., 1999; Schilling et al., 1999; Wheeler et al., 2000). Therefore, proteolytic processing of mutant Htt is assumed to play a key role in the pathogenesis of HD.

The age-related decline in protein homeostasis challenges the capacity of neurons to counteract the accumulation of misfolded proteins, such as Htt fragments, and this may explain in part the late onset of HD and other protein conformation diseases (Balch et al., 2008). However, the role of inclusions in HD has been challenged and inclusions have even been thought to be protective (Arrasate et al., 2004; Arrasate and Finkbeiner, 2012). Interestingly, neuronal Htt aggregates found in human postmortem brain material are positive for ubiquitin, indicating a role of the ubiquitin-proteasome related protein quality control system (DiFiglia et al., 1997; Gutekunst et al., 1999; Sieradzan et al., 1999). Previous studies showed that wildtype and mutant Htt are cleared by the proteasomal and autophagosomal pathways (Wyttenbach et al., 2000; Waelter et al., 2001; Qin et al., 2003; Thompson et al., 2009; Li et al., 2010; Juenemann et al., 2013). Furthermore, Htt can be ubiquitinated at its N-terminal region, suggesting specific ubiquitin-mediated degradation by cellular clearance mechanism (Steffan et al., 2004; Juenemann et al., 2013; Lu et al., 2013; Bhat et al., 2014).

Covalent attachment of ubiquitin to a lysine side chain of a target protein is a multistep process. Different enzymatic components are required for protein ubiquitination, including ubiquitin-activating enzyme (E1), ubiquitin-conjugating enzyme (E2), and ubiquitin-ligating enzyme (E3) (Hershko and Ciechanover, 1998). Ubiquitin itself contains seven lysine residues that can be modified by successive rounds of ubiquitination resulting in isopeptide-linked ubiquitin chains of distinct topologies determining the cellular purpose of the ubiquitinated substrate. Protein ubiquitination is important for diverse intracellular processes such as degradation and localization (Komander and Rape, 2012). Previous studies have shown that Htt can be ubiquitinated via the action of specific E3 ubiquitin ligases. Transient overexpression of the co-chaperone and E3 ligase C-terminus of HSC70-interacting protein (CHIP) increases the ubiquitination and clearance of polyQ-expanded Htt and ataxin-3 in a cell culture model (Jana et al., 2005). Overexpression of Ube3a in an HD knock-in mouse model enhances mutant Htt degradation via the proteasomal pathway resulting in reduced Htt aggregation (Bhat et al., 2014). Furthermore, other E3 ubiquitin ligases, such as ERAD-associated E3 ubiquitin-protein ligase (HRD1), TNF receptor-associated factor 6 (TRAF6), and ubiquitin-like with PHD and ring finger domains (UHRF-2), are supposed to ubiquitinate Htt independent of the polyQ-length (Yang et al., 2007; Iwata et al., 2009; Zucchelli et al., 2011).

Studying the intracellular ubiquitination pattern of soluble and insoluble mutant N-terminal Htt fragments offers a valuable insight into Htt aggregation, and reveals whether Htt is properly ubiquitinated for proteasomal or autophagosomal degradation.

To study the composition of Htt species in aggregates solubilization thereof is mandatory. Hazeki et al. showed that Htt aggregates occuring in transfected COS cells overexpressing mutant Htt-exon1 can be solubilized by pure formic acid into monomeric forms (Hazeki et al., 2000). High molecular weight complexes of mutant Htt dissociate in concentrated formic acid and HCl, whereas 8M Urea, 6M Guanidine/HCl, 1N NaOH and pure acedic acid showed low potential to interfere with Htt complex stability. In addition, Iuchi et al. showed that inclusions in PC12 cells formed by polyQ-stretches of the mutant androgen receptor are dissolved to monomers by the use of concentrated formic acid (Iuchi et al., 2003). Dissociation of inclusion bodies in T-hd122 cells expressing truncated mutant Htt revealed ubiquitination of the Htt proteolytic cleavage fragment cp-A (Lunkes et al., 2002).

Here we describe protocols for solubilization of Htt aggregates, based on sodium dodecyl sulfate (SDS) and formic acid treatment, which allow to identify post-translational modifications such as ubiquitination. The described methods for solubilization of intracellular inclusion bodies composed of mutant Htt N-terminal fragments can be potentially transferred in order to study further neurodegenerative diseases.

## Material and methods

### Constructs

The Htt-exon1-97Q construct was generated by replacing the C-terminal green fluorescent protein (GFP) sequence of Htt-exon1-97Q-GFP (kindly provided by R. Kopito, Stanford University, USA) for a stop codon and the Htt-exon1-97Q-H4 construct was generated by cloning the Htt-exon1-97Q sequence with a 5’ *XhoI* and 3’ *BamHI* site into a vector encoding a C-terminal H4-tag (His-HA-HA-His, kindly provided by J. Steffan, University of California, USA). The reporter construct Htt-exon1-25Q-GFP was kindly provided by R. Kopito and the ubiquitin constructs HA-Ub-wt and HA-Ub-K0 were a kind gift from N. Zelcer (Academic Medical Center, University of Amsterdam, The Netherlands).

### Cell culture and transfection

Neuro-2a cells were maintained in Dulbecco’s modified Eagle’s medium (DMEM) (Invitrogen) supplemented with 10% fetal calf serum, 1 mM glutamine, 100 U/mL penicillin, and 100 μg/mL streptomycin in a humidified incubator with 5% atmospheric CO_2_. Neuro-2a cells were seeded in 6-well plates and transfected with polyethylenimine according to the manufacturer’s instructions (Polysciences Europe).

### Western blot analysis of Triton X-100 soluble proteins

Neuro-2a cells were harvested in lysis buffer (50 mM Tris/HCl pH 7.4, 150 mM NaCl, 1 mM EDTA, 1% Triton X-100, 20 mM NEM, supplemented with complete mini protease inhibitor cocktail (Roche)) 24 h after transfection. Total cell lysate was boiled for 10 min at 99°C with 1x laemmli sample loading buffer (350 mM Tris/HCl pH 6.8, 10% SDS, 30% glycerol, 6% β-mercaptoethanol, bromphenol blue), fractionated on a 12% SDS-gel by SDS-PAGE gel electrophoresis and transferred to a polyvinylidene fluoride (PVDF) membrane (0.45 μm pore size, Schleicher and Schuell). Western blot membranes were blocked with 5% milk, incubated with primary antibodies anti-Htt 1C2 (1:1000, Millipore, MAB1574), anti-Htt N18 (1:1000, Enzo, BML-PW0595-0100), anti-HA (1:1000, Sigma- Aldrich, H3663), anti-β-actin (1:1000, Santa Cruz, SC-130656), and anti-ubiquitin (1:100, Sigma-Aldrich, U5379), and subsequently incubated with secondary antibodies IRDye 680 or IRDye 800 (1:10,000; LI-COR Biosciences). Infrared signal was detected using the Odyssey imaging system (Licor).

### Filter trap assay

For analysis of Triton X-100-insoluble aggregates, filter trap assay was performed with the pellet obtained after centrifugation of the Triton X-100 cell lysate (15 min at 14,000 rpm at 4°C). Pellet with aggregates was resuspended in benzonase buffer (1 mM MgCl_2_, 50 mM Tris/HCl pH 8.0) and incubated for 1 h at 37°C with 125 U Benzonase (Merck). Reaction was stopped with 2x termination buffer (40 mM EDTA, 4% SDS, 100 mM DTT). Samples with a protein concentration of 50 μg were diluted in 2% SDS buffer and filtered through a 0.2 μm pore size cellulose acetate membrane (Schleicher and Schuell), pre-equilibrated in 2% SDS wash buffer (2% SDS, 150 mM NaCl, 10 mM Tris/HCl pH 8.0) and spotted on the membrane in doublets. Filters were washed twice with 0.1% SDS buffer (0.1% SDS, 150 mM NaCl, 10 mM Tris pH 8.0) and blocked with 5% milk for further treatment similar to western blot membranes.

### Fluorescence microscopy

Neuro-2a cells were seeded on coverslips in a 6-well plate and transfected with the indicated DNA constructs 24 h prior to imaging. Transfected cells were fixed with 4% paraformaldehyde (PFA) and images were obtained using a confocal microscope equipped with an Ar/Kr laser and a 63x objective (Leica TCS SP8).

### SDS-soluble/insoluble fractionation for analysis of post-translational modifications

#### Procedure

1. Cells grown in a 6-well plate were washed with 1 mL ice cold 1x PBS and harvested in 0.5 mL ice cold 1x PBS with a cell scraper.
2. The cell suspension was transferred to a 1.5 mL tube and centrifuged for 5 min (2000 rpm, 4°C).
3. After discarding the supernatant, cells were lysed by adding 0.1 ml 1x TEX buffer directly on top of the cell pellet without pipetting up and down (cell pellet should get loose from the tube wall) followed by immediate high speed vortexing for 5–10 s.
4. Sample was sonicated with a benchtop Ultrasonic Disintegrator (Soniprep150, Sanyo) using a 10 s pulse until the sample got liquid and was not slurry anymore.
5. In addition, the DNA was sheared by passing the sample solution 6 times through a 21 Gauge needle.
6. The proteins were reduced by adding 5 μl 1 M DTT (fresh) to the sample and subsequently boiled for 10 min at 99°C in a thermoblock while shaking at 1000 rpm.
7. Afterwards the sample was immediately centrifuged for at least 60 min (14,000 rpm, RT).
8. Maximal four samples at once were taken out of the centrifuge and the remaining samples were continuously centrifuged until further use.
9. The supernatant (SDS-soluble fraction) was carefully transferred into a new tube containing 5 μl concentrated bromphenol blue solution (soluble fraction) carefully avoiding to touch the SDS-insoluble pellet.
  **!Caution**: The SDS-insoluble pellet is invisible and detaches very easily from the tube wall.
10. For solubilization of the SDS-insoluble aggregates, 10 μl 100% formic acid was added to the pellet and pipetted several times up and down.
11. The suspension was incubated for 40 min at 37°C while shaking at 1000 rpm in a thermoblock.
12. Then the formic acid was evaporated overnight at 30°C using a Speedvac system (Eppendorf).
13. The remaining protein pellet was solved in 50 μl 1x TEX blue buffer by pipetting up and down (SDS-insoluble fraction).
  **!Caution**: If the formic acid is not fully evaporated after step 12 the sample will be still acidic and the bromphenol blue indicator will switch color from blue to green/yellow. In this case, the sample can be neutralized by adding 5 μl 1 M Tris/HCl pH 8.0.
14. The sample was boiled for 10 min at 99°C while shaking at 1000 rpm.
15. To visualize formic acid-dissolved aggregates by western blot analysis 25–50 μl of the SDS-insoluble fraction was loaded on a 12% SDS-PAGE gel.
  **!Caution**: To compare several SDS-insoluble fractions, equivalent volumes have to be loaded.
16. Not immediately used sample fractions were stored at 4°C.

#### Buffers

**Table:** 

1x PBS:	137 mM NaCl, 2.7 mM KCl, 4.3 mM Na^2^PO^4^, 1.4 mM KH^2^PO^4^, adjust to pH 7.4
1x TEX buffer:	70 mM Tris/HCl pH 6.8, 1.5% SDS, 20% glycerol (store at RT)
1x TEX buffer blue:	70 mM Tris/HCl pH 6.8, 1.5% SDS, 20% glycerol, tip of a spatula of bromphenol blue (store at RT)
Concentrated bromphenol blue solution:	spatula-tip of bromphenol blue solved in water (store at RT)

## Results and discussion

**Figure 1:**
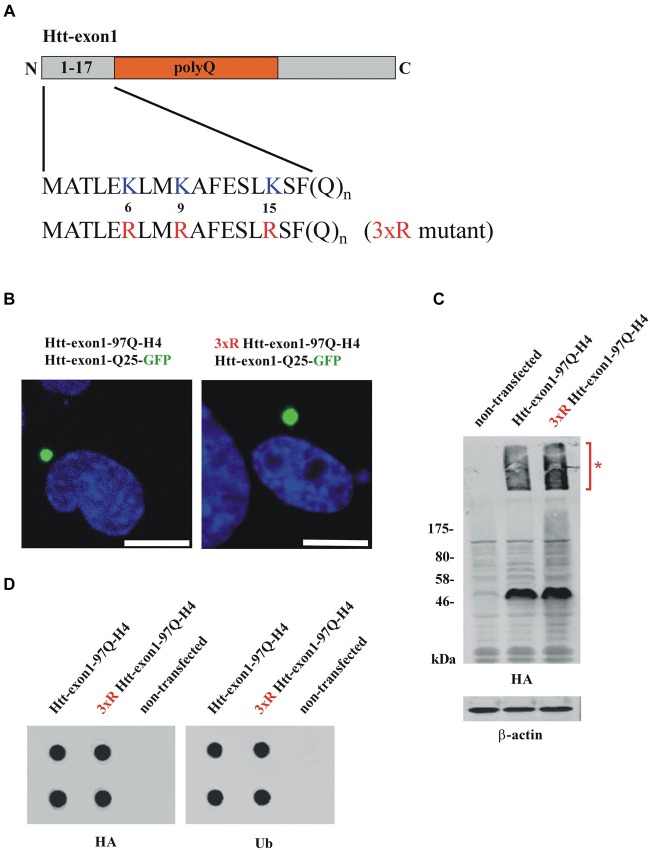
**Conventional methods to study Htt protein aggregates. (A)** Scheme of the Htt-exon1 protein fragment. N-terminal region (aa 1-17) of wildtype Htt-exon1 with three lysine residues (K6, K9, K15) compared to the Htt mutant (3xR) containing three arginine residues (R6, R9, R15). **(B)** Aggregates of wildtype and 3xR mutant Htt-exon1. Transient co-transfection of Neuro-2a cells with the aggregation reporter Htt-exon1-25Q-GFP and wildtype or 3xR mutant Htt-exon1-97Q-H4 constructs. After 24 h cells were fixed and aggregates of Htt-exon1 co-sequestering the aggregation reporter Htt-exon1-25Q-GFP were detected by fluorescence of GFP. The nucleus was stained by 4’,6-diamidino-2-phenylindole (DAPI). Scale bar: 10 μm. **(C)** Western blot analysis and **(D)** filter trap assay (in doublets) of cell lysates from Neuro-2a cells transient transfected for 24 h with wildtype and 3xR mutant Htt-exon1-97Q-H4 constructs. Triton X-100-soluble Htt-exon1 proteins and insoluble Htt aggregates (asterisk) in the stacking gel were detected on immunoblots with HA antibody. β-actin was used as loading control. Filter trap assay was performed using an antibody against the HA-tag of Htt and an antibody against ubiquitin (Ub) recognizing ubiquitinated proteins.

Htt contains three lysine residues (K6, K9, K15) at the N17 region for putative ubiquitination (Figure 1A). Previous work from Steffan et al. has shown that mutation of these three lysines to arginines obviates ubiquitination and SUMOylation of soluble Htt-exon1 in cell culture (Steffan et al., 2004). An AQUA-MS approach revealed that less than 1% of soluble N-terminal Htt expressed in HEK 293 cells is ubiquitinated without considering inhibition of cellular degradation pathways (Hipp et al., 2012). Immunoprecipitation and mass spectrometry analysis are appropriate methods to identify specific soluble protein-ubiquitin conjugates. However, once misfolded proteins start to aggregate and interacting partners are co-sequestered, this represents a challenge in terms of isolation and detection of specific protein-ubiquitin conjugates.

In the last two decades, various methods have been established to detect and analyze aggregates of misfolded proteins related to neurodegeneration. Htt with an expanded polyQ-stretch is prone to misfold, triggering its aggregation, a property tightly associated with its neurotoxicity (Takahashi et al., 2010; Miller et al., 2011). Htt can be found *in vitro* and *in vivo* in a monomeric or aggregated state, and they are both detectable with different methods, including microscopic techniques, fluorescence-activated cell sorting (FACS) analysis or biochemical approaches. Until now, these methods are not applicable for analyzing post-translational modified Htt species trapped in aggregates, especially with respect to distinguishing between Htt-ubiquitin conjugates and Htt that is non-covalently associated with monoubiquitin, polyubiquitin conjugates or other co-sequestered polyubiquitinated proteins.

To investigate the connection between ubiquitination and intracellular aggregation of Htt, cells overexpressing Htt-exon1-97Q and as a control a mutant form lacking three N-terminal lysine residues (3xR mutant) necessary for Htt ubiquitination were analyzed by fluorescence microscopy, western blot analysis and filter trap assay. To study whether aggregation of Htt depends on its ubiquitination Neuro-2a cells were co-transfected with either Htt-exon1-97Q or its 3xR mutant variant and the aggregation reporter Htt-exon1-25Q-GFP, respectively, and analyzed using confocal fluorescence microscopy (Figure 1B). The aggregation reporter Htt-exon1-25Q-GFP shows a diffuse cellular staining unless sequestered into aggregates (Juenemann et al., 2013). Both wildtype and 3xR mutant Htt-exon1-97Q form inclusion bodies within 24 h after transfection as detected with the GFP-tagged Htt-exon1-25Q reporter protein that becomes sequestered into the mutant Htt-exon1-Q97 aggregates, indicating that Htt aggregation is independent of its ubiquitination. To evaluate whether Htt aggregates are detectable by western blot Triton X-100 soluble fractions of cells transfected with Htt-exon1-97Q-H4 or 3xR mutant Htt-exon1-97Q-H4 were separated by SDS-PAGE. Both Triton X-100 insoluble Htt aggregates in the stacking gel (asterisk) and the soluble Htt-exon1 monomer were recognized by using a mouse monoclonal HA antibody (Figure 1C). The Triton X-100 insoluble aggregates of the same transfected Neuro-2a cells were applied to a filter trap assay. The filter trap membrane was stained against Htt-exon1-97Q-H4 and ubiquitin using an HA and a ubiquitin antibody (Figure 1D). Both wildtype and 3xR mutant Htt-exon1-97Q aggregates showed a positive ubiquitin staining, which could either mean that the three Htt N-terminal lysine residues are not essential for ubiquitination, there is aggregate association with ubiquitin or other ubiquitinated proteins are co-sequestered.

Together, this indicates that with microscopy, immunoblot and filter trap assay there is no discrimination between ubiquitin covalently bound to aggregated Htt-exon1 and non-covalently associated monoubiquitin, polyubiquitin conjugates or sequestered polyubiquitinated proteins. To identify ubiquitinated Htt species besides monomeric Htt within aggregates an adequate solubilization is required that is capable of destroying the strong non-covalent interactions between the aggregated proteins.

**Figure 2:**
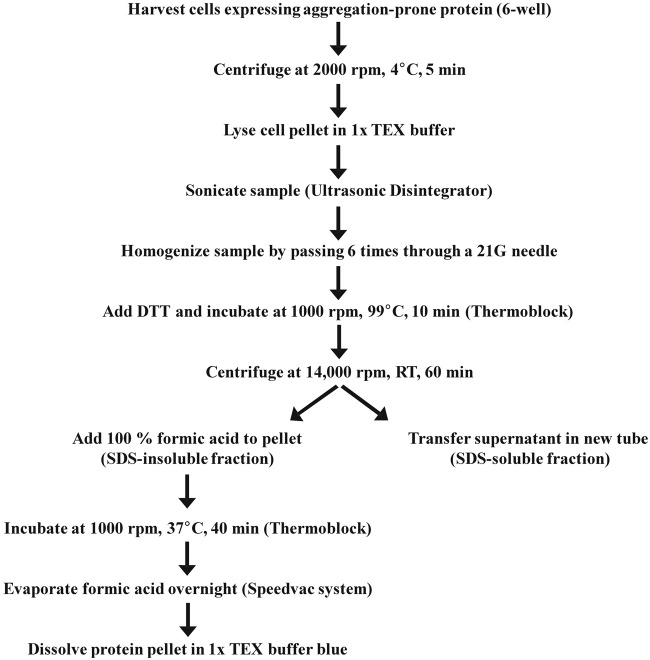
**A stepwise protocol of SDS-soluble and SDS-insoluble cell fractionation**.

Here we provide a protocol suitable for subsequent biochemical protein analysis of aggregates, consisting of mutant Htt, by solubilizing them with 100% formic acid (Figure 2). Formic acid disrupts protein interactions such as hydrogen bonds. Previous work indicated that aggregation of Htt results from the formation of hydrogen bonds between polyQ stretches forming β-sheet structures (Perutz et al., 1994). Hazeki et al. showed that only formic acid and HCl but not acedic acid, Urea, Guanidine/HCl or NaOH dissociate higher molecular weight complexes of mutant Htt (Hazeki et al., 2000).

**Figure 3:**
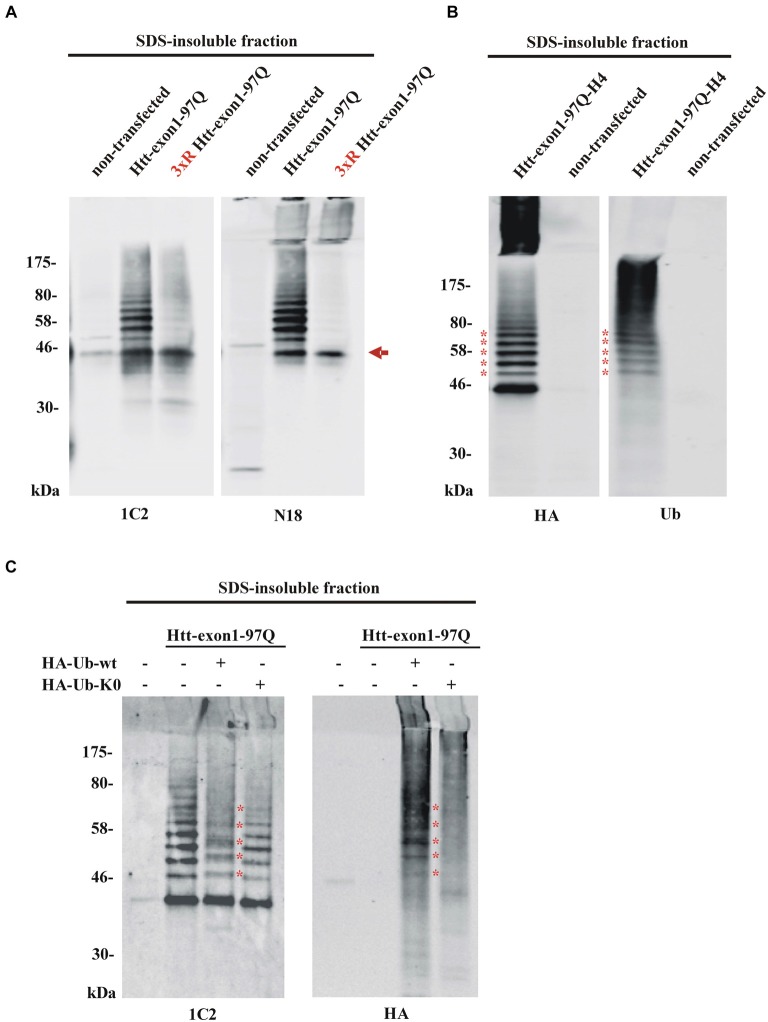
**Detection of aggregated Htt and its ubiquitinated species. (A)** SDS-insoluble fraction of Neuro-2a cell lysate after transient transfection of cells with wildtype or 3xR mutant Htt-exon1-97Q constructs. Formic acid-dissolved aggregates of Htt were detected on western blot by the Htt-specific antibodies 1C2 and N18. Formic acid soluble Htt-exon1 monomer (arrow) and a higher wildtype Htt-specific protein ladder, which is not detectable with 3xR mutant Htt, are shown. Remaining non-dissolved Htt aggregates were trapped in the stacking gel and recognized by the N18 antibody only. **(B)** Western blot analysis of the SDS-insoluble fraction of Neuro-2a cells transiently transfected with Htt-exon1-97Q-H4. In addition to the monomeric Htt-exon1-97Q-H4 protein a ubiquitin-positive Htt protein ladder (asterisks) is shown by the HA and ubiquitin (Ub) antibody, respectively. **(C)** Western blot analysis of the SDS-insoluble fraction of Neuro-2a cells transiently co-transfected with Htt-exon1-97Q and the ubiquitin constructs HA-Ub-wt and its lysine-dead mutant HA-Ub-K0, respectively. In addition to the monomeric Htt-exon1-97Q protein a ubiquitin-positive Htt protein ladder is shown by the 1C2 and Ub antibody, respectively. Lysine residue-dependent integration of HA-Ub-wt into the Htt-exon1 polyubiquitination chains is shown by an upward shift of the ubiquitin-modified Htt bands at the size of the ubiquitin N-terminal HA-tag (asterisks).

In order to solubilize SDS-insoluble protein pellets containing aggregated Htt from Neuro-2a cells transfected with the constructs encoding Htt-exon1-97Q and its 3xR Htt mutant variant, pellets were treated with 100% formic acid followed by SDS-PAGE and immunoblot analysis. The formic acid treated SDS-insoluble fraction showed the presence of monomeric Htt-exon1-97Q at around 45 kDa (arrow) and at higher molecular weight post-translational modified species of Htt-exon1-97Q in form of distinct bands (Figure 3A). Htt was stained by the Htt-specific antibodies 1C2 against the polyQ tract and N18 against the N-terminal region. With the 3xR Htt mutant variant the higher molecular Htt bands disappeared, indicating that the post-translational modification of Htt is dependent on the N-terminal lysine residues. The size difference between the distinct bands suggests a ubiquitination of Htt. The observation that the aggregated 3xR Htt mutant does not show this high molecular weight bands indicates that the Htt N-terminal lysine residues are the ubiquitination sides and not, as described as more rare forms of ubiquitination, serine, threonine and cysteine residues or the N-terminal α-amino group ubiquitination in a linear fashion (McDowell and Philpott, 2013).

To confirm that the higher molecular weight protein ladder of formic acid-dissolved HA-tagged Htt-exon1-97Q indeed represents polyubiquitinated Htt, membranes were stained in parallel with an HA antibody and a ubiquitin antibody. Both antibodies detect the same protein bands indicating that the specific bands are ubiquitinated species of Htt-exon1 (asterisks) (Figure 3B). Worth mentioning is the difference between the used antibodies regarding the recognition of formic acid-nondissolved aggregates in the SDS stacking gel. The Htt specific N-terminal polyclonal N18 antibody and the HA antibody against the C-terminal HA-tag of the Htt protein compared to the Htt-specific monoclonal 1C2 antibody and the ubiquitin antibody are capable of detecting higher molecular Htt aggregates in the SDS stacking gel. The solubilization of Htt aggregates by pure formic acid is incomplete, since aggregates are still detectable by certain antibodies in the stacking gel. This might be a consequence of high Htt accumulation due to overexpression and part of the aggregates are not solved in time, or of the potentially formic acid-resistant aggregated Htt species. Previous studies suggested that aggregated Htt is crosslinked by covalent bonds such as those formed by transglutaminase (Kahlem et al., 1996; Cooper et al., 2002; Iuchi et al., 2003; Zainelli et al., 2003, 2004).

To validate Htt ubiquitination in cellular aggregates, SDS-insoluble Htt from Neuro-2a cells co-transfected with Htt-exon1-97Q and either HA-tagged Ub-wt or Ub-K0, where all the lysine residues are exchanged to an arginine to prevent ubiquitination, was analyzed. Incorporation of HA-Ub into the Htt polyubiquitin chain is indicated by shifts of each Htt-Ub bands upwards in the size of one (about 1 kDa, asterisks) or more HA-tags creating indistinct protein bands as detected by the 1C2 antibody (Figure 3C). Co-staining with the HA antibody confirms covalent binding of the overexpressed HA-Ub-wt to the Htt-exon1 protein and its ubiquitinated species (asterisks) showing that Htt gets post-translational modified by ubiquitin. However, co-expression of HA-Ub-K0 reveals no incorporation of ubiquitin lacking lysine residues.

Here we provide a protocol for the solubilization of aggregated Htt proteins using formic acid to detect ubiquitinated species of overexpressed mutant Htt-exon1. Whether this method is sensitive enough to detect ubiquitination of endogenous expressed Htt depends on the quantity of aggregated Htt and the rate of Htt ubiquitination/deubiquitination. The presented methods might be transferrable to study other neurodegenerative diseases besides HD that are hallmarked by misfolded proteins prone to aggregate. In addition, the protocol may be applied to analyze other post-translational modifications of misfolded proteins in SDS-insoluble aggregates, such as SUMOylation, phosphorylation or acetylation.

While studying the role of ubiquitination of a specific misfolded protein within the cell it is important to distinguish between the analyzed misfolded protein covalently binding ubiquitin and ubiquitin only associated with the aggregate such as co-sequestered polyubiquitinated proteins.

Further research and the identification of the ubiquitin linkage pattern of soluble and insoluble Htt dependent on the length of the polyQ stretch or cellular localization is important to understand the fate of this protein within the cell. Unraveling the disease-related alterations of Htt post-translational modifications will certainly fill a crucial gap between the current knowledge of Htt fragment generation and aggregation on the one side, and regulation of potent degradation machineries on the other side that are capable of obviating toxicity of Htt species within the cell.

## Conflict of interest statement

The authors declare that the research was conducted in the absence of any commercial or financial relationships that could be construed as a potential conflict of interest.
